# Scapular kinematics and muscle activity during Yi Jin Bang exercises

**DOI:** 10.3389/fphys.2023.1169092

**Published:** 2023-06-08

**Authors:** Jinde Liu, Stanley Sai-chuen Hui, Yijian Yang, Yanhao Liu, Qipeng Song, Dewei Mao

**Affiliations:** ^1^ Department of Sports Science and Physical Education, The Chinese University of Hong Kong, Hong Kong, China; ^2^ College of Sports and Health, Shandong Sport University, Jinan, China

**Keywords:** electromyography, motion analysis, rehabilitation, scapula, shoulder

## Abstract

**Introduction:** Scapular dyskinesis is commonly associated with subacromial pain syndrome (SAPS). Addressing scapular dyskinesis is widely accepted as an important component of shoulder rehabilitation. Our previous randomized controlled trial showed that Yi Jin Bang (YJB) exercises could effectively manage SAPS, but scapular motions and muscle activity during YJB exercises remain unknown. This study examined scapular kinematics synchronously with scapular muscle activation during YJB exercises.

**Methods:** Thirty healthy participants with no shoulder complaints were enrolled in this study. Three-dimensional (3D) scapular kinematics and electromyography (EMG) activation of the upper trapezius, middle trapezius, lower trapezius, serratus anterior, anterior deltoid, middle deltoid, and posterior deltoid were synchronously measured during nine YJB movements.

**Results:** During all YJB movements, the scapula was upwardly rotated and anteriorly tilted, with more upward rotation and a similar or less anterior tilt than the mean resting scapular angle. Column rotation, arm crossover, shoulder support circle, and armpit support high lift generated more internal rotation than the mean resting scapular angle, with the angles of internal rotation significantly greater than the other five movements (*p* < 0.001). Regarding EMG activity, all YJB movements elicited low activity (1.42%–19.19% maximal voluntary isometric contraction [MVIC]) from the upper trapezius and posterior deltoid and low to moderate activity (0.52%–29.50% MVIC) from the middle trapezius, lower trapezius, serratus anterior, anterior deltoid, and middle deltoid.

**Conclusion:** YJB exercises could be useful in the middle to later phases of shoulder rehabilitation. For patients with insufficient external rotation, some YJB movements should be prescribed with caution.

## 1 Introduction

Subacromial pain syndrome (SAPS) is the most common type of shoulder pain that can affect any age group but tends to be more common in older people or people engaged in repetitive overhead activities (Chipchase et al., 2000; Kinsella and Pizzari, 2017). The SAPS encompasses a large group of shoulder problems (e.g., bursitis, tendinitis calcarea, supraspinatus tendinopathy, partial tear of the rotator cuff, biceps tendinitis, and tendon cuff degeneration) that cause pain localized around the acromion, often exacerbating while performing repetitive work at or above shoulder level (Diercks et al., 2014; Haik et al., 2016; Reijneveld et al., 2017). Although the pathogenesis of SAPS is often debated, scapular dyskinesis (i.e., alterations in normal scapular motion and position) is an important risk factor for SAPS (Cools et al., 2007; Kibler et al., 2013; Reijneveld et al., 2017; Park et al., 2020). Scapular dyskinesis may result from alterations in scapular muscle neuromuscular control, including hyperactivity of the upper trapezius muscles and reduced activity of the middle trapezius, lower trapezius, and serratus anterior muscles (Ludewig and Cook, 2000; Lin et al., 2005; Ludewig and Braman, 2011).

Electromyography (EMG) has been widely used by clinicians and investigators to examine muscle activity during rehabilitation exercises (Berckmans et al., 2020). Based on the findings of altered activation in the scapular muscles for individuals with SAPS, therapeutic exercises designed to improve scapular control should focus on promoting the activity of the middle trapezius, lower trapezius, and serratus anterior muscles while reducing the activity of the upper trapezius muscles (De Mey et al., 2012; Huang et al., 2013). Hyperactivity of the deltoid has been suggested to narrow the subacromial space due to superior translation of the humeral head during arm elevation relative to the glenoid fossa, which may increase the risk of SAPS (Tsuruike and Ellenbecker, 2019). Thus, the deltoid muscle activity during shoulder and scapular exercises should be considered to avoid over-recruitment of the deltoid muscle. In addition, the alterations in scapular kinematics of patients with SAPS are often characterized by decreasing scapular upward rotation and increasing scapular internal rotation and anterior tilt (Kibler et al., 2008; Struyf et al., 2011; Camargo et al., 2015; Grimes et al., 2019). The information on three-dimensional (3D) scapular motion relative to the thorax during shoulder exercises could provide additional helpful information for selecting exercises for a rehabilitation program (Berckmans et al., 2020).

Exercise therapy has been demonstrated as the best conservative therapy to improve pain, function, and range of motion in individuals with shoulder pain (Haik et al., 2016; Liu et al., 2022). Good adherence to exercise treatment is an important factor that can influence the effectiveness of treatment (Deutscher et al., 2009). Since lack of time, convenience, and financial constraints are the main sources of poor adherence, an easy-to-learn and perform exercise is assumed to be beneficial for good adherence (Jack et al., 2010). Yi Jin Bang (YJB) is a novel home-based Chinese mind-body Qigong exercise consisting of nine easy-to-learn movements performed with the aid of a 24–27 inches long baton (see Supplementary Material S1 for more details of the nine YJB movements) (Hui et al., 2022). Our previous randomized controlled trial showed that 10 weeks of YJB exercises have similar effects as traditional exercise therapy (i.e., stretching and strengthening exercises) in reducing pain and disability and improving mobility in individuals with SAPS (Hui et al., 2022). Compared with traditional exercise therapy, YJB exercises require only a small space and simple equipment to practice at home. Patients may find the nine movements routine of YJB much easier to learn and follow, thus increasing compliance and decreasing the number of clinical supervisions. However, the underlying biomechanical mechanism for the training effects of YJB exercises on SAPS remains unclear. Since the scapula plays different crucial roles in normal shoulder function, scapular-based exercises should be an inherent part of a SAPS rehabilitation program (Berckmans et al., 2021). The purpose of this study was to examine the 3D scapular kinematics synchronously with the EMG activation of the scapular stabilizers during YJB exercises. We hypothesized that there would be differences in 3D scapular motion and in the EMG amplitude of scapular stabilizers between different YJB movements.

## 2 Methods

### 2.1 Participants

The participants were recruited from Shandong Sport University through leaflets posted on campus. Participants were included if they met the following criteria (Kinsella and Pizzari, 2017): aged 18–40 years (Chipchase et al., 2000); had no shoulder or neck complaints in the past 6 months (Diercks et al., 2014); had no surgery, fracture, or dislocation of the shoulder in the past (Reijneveld et al., 2017); scored below 25 on the simplified Chinese version of the Disabilities of the Arm, Shoulder and Hand (DASH) questionnaire. The DASH is a 30-item self-report questionnaire designed to measure symptoms and physical function related to the arm, shoulder, or hand, scored from 0 (no disability) to 100 (most severe disability) (Jester et al., 2005). The simplified Chinese version of DASH has proven valid and reliable for measuring patients with upper extremity disorders (Chen et al., 2015). Participants were excluded if they had any neurological disorders or medical restrictions on physical activity.

### 2.2 Sample size estimation

Sample size estimation was performed using G*Power 3.1. Using the calculated Cohen’s d of one for EMG activity based on the minimal relevant difference of 10% in EMG activity (estimated SD of 10%) and the calculated Cohen’s d of 0.56 for 3D scapular kinematics based on the minimal relevant difference of 5° in 3D scapular kinematics (estimated SD of 9°), 27 participants were needed to achieve 80% statistical power with *α* = 0.05 (Ludewig and Cook, 2000; Berckmans et al., 2020).

### 2.3 Study design

This laboratory-based, cross-sectional study was conducted according to the STROBE statement (von Elm et al., 2008). The 3D kinematics of the scapular rotations were measured and synchronized with the EMG data of four scapulothoracic muscles (upper trapezius, middle trapezius, lower trapezius, and serratus anterior) and three scapulohumeral muscles (anterior deltoid, middle deltoid, and posterior deltoid) on the dominant side during nine movements of the YJB exercise. A researcher determined the dominant arm by asking each participant which arms they used to perform well-learned skills such as throwing. The participants randomly performed each movement three times, with 30-s rest between repetitions and 60-s rest between movements. Each movement was isometrically held in the last phase for 5 s, with speed controlled using a metronome (set at 60 beats/min). Before formal testing, the participants learned and practiced the YJB exercise under the one-to-one guidance of a certified YJB instructor. In addition, the YJB instructor also led each participant to perform each movement and provided verbal movement correction cues during formal testing. The 3D kinematic and EMG data collected during the 5-s isometric hold of each movement were used for data analysis.

### 2.4 Procedures

**FIGURE 1:**
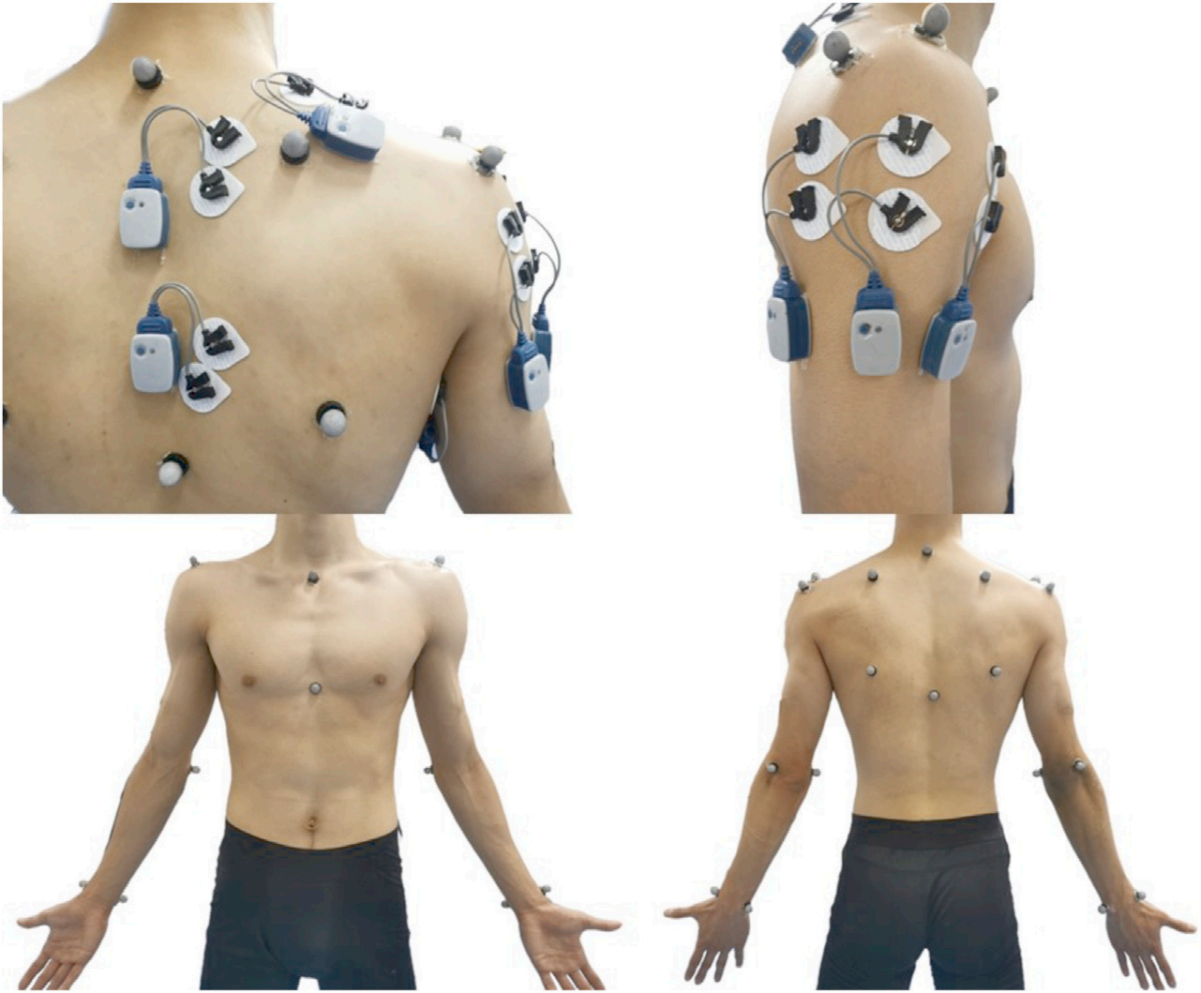
The location of the EMG and the marker set.

Before electrode placement, the skin was prepared by shaving any hair overlying it and cleaning it with alcohol wipes to reduce skin impedance. Bipolar silver/silver chloride surface electrodes were placed at a 2-cm interelectrode distance over four scapulothoracic muscles (upper trapezius, middle trapezius, lower trapezius, and serratus anterior) and three scapulohumeral muscles (anterior deltoid, middle deltoid, and posterior deltoid). Electrodes were placed on the muscle belly of each muscle along with the orientation of the muscle fibers, according to the recommendation of Criswell (Figure 1) (Criswell, 2011). To ensure consistency with electrode placement, only one researcher placed all electrodes throughout the study. EMG data were collected using a wireless Noraxon TeleMyo Direct Transmission System (Noraxon Inc., AZ, United States) at a sampling rate of 2,000 Hz. A senior researcher verified the correct electrode placement and the quality of the EMG signal by inspecting the EMG signal while performing specific submaximal isometric contractions.

Before the maximal voluntary isometric contraction (MVIC), participants performed a warm-up consisting of multidirectional shoulder movements. The participants performed the MVIC in manual muscle test positions specific to each muscle of interest. The MVIC testing procedures for all seven muscles are presented in Table 1 (Schuldt and Harms-Ringdahl, 1988; Kendall et al., 2015; Cools et al., 2020; Werin et al., 2020). Three 5-s MVIC trials were conducted for each muscle, with a 30-s rest between repetitions and a 2-min rest between muscles. The duration of the contraction was controlled using a metronome. A researcher provided the participants with verbal and visual feedback to promote maximal exertion.

**TABLE 1:** Description of maximal voluntary isometric contractions testing procedure for the seven muscles of interest.

Muscle	Testing procedure
Upper trapezius	Seated position, both feet were supported, and the tested arm was abducted at 90°. Resistance was applied proximal to the elbow in the downward direction (to resist further abduction)
Middle trapezius	Prone position, the head was rotated to the contralateral side. The contralateral arm was abducted at 45°, and the tested arm was abducted at 90° in maximal external rotation (thumb up). Resistance was applied proximal to the elbow in the downward direction (to resist further horizontal abduction)
Lower trapezius	Prone position, the head and the contralateral arm were held in the same position as for middle trapezius. The tested arm was abducted at 145° in maximal external rotation (thumb up). Resistance was applied proximal to the elbow in the downward direction (to resist further elevation)
Serratus anterior	Seated position, both feet were supported, and the tested arm was extended and flexed at 135°. Resistance was applied proximal to the elbow in the downward direction (to resist further forward flexion)
Anterior deltoid	Seated position, both feet were supported, and the tested arm was placed in abduction, slight flexion, and slight external rotation. Resistance was applied on the anterolateral surface of the arm in the direction of adduction and extension (to resist further abduction and flexion)
Middle deltoid	Seated position, both feet were supported, and the tested arm was placed in the abduction and neutral rotation. Resistance was applied on the dorsal aspect of the distal end of the humerus in the direction of adduction (to resist further abduction)
Posterior deltoid	Seated position, both feet were supported, and the tested arm was placed in abduction, slight extension, and slight internal rotation. Resistance was applied on the posterolateral surface of the arm in the direction of adduction and flexion (to resist further abduction and extension)

After performing the MVIC, a 3D motion capture system (Vicon, Oxford Metrics Ltd., Oxford, United Kingdom) with 12 cameras was used to assess the 3D scapular resting position during a standing posture with the arms relaxed at the side, followed by collecting the 3D scapular kinematic data during the YJB exercise. The motion was filmed at 200 Hz after standardized calibration procedures. Reflective markers were placed over anatomical landmarks at the thorax (seventh cervical vertebra, eighth thoracic vertebra, jugular notch, and xiphoid process), bilateral scapula (trigonum spinae scapulae, inferior angle, acromial angle, and coracoid process), bilateral humerus (lateral and medial epicondyles), and bilateral forearm (radial and ulnar styloids) as per the recommendations of the International Society of Biomechanics (Figure 1) (Wu et al., 2005). The glenohumeral joint center was determined by performing a 10-s shoulder circumduction trial (Monnet et al., 2007). The EMG and 3D kinematics data were collected synchronously using Nexus software (Vicon, Oxford Metrics Ltd., Oxford, United Kingdom) during the performance of the YJB exercise.

### 2.5 Data processing

The raw kinematic data collected from the scapular were exported as C3D files and uploaded to Visual 3D software (C-motion Inc., MD, United States) for further processing. Scapular motions were calculated as the Euler angles of the scapula relative to the thorax reference frames following the Y-X-Z sequence, with the motion defining external/internal rotation around the *Y*-axis, upward/downward rotation around the *X*-axis, and anterior/posterior tilt around the *Z*-axis (Wu et al., 2005). The scapular kinematic data were smoothed through a fourth-order Butterworth low-pass digital filter at an estimated optimum cutoff frequency of 6.6 Hz (Thigpen et al., 2006). The EMG data processing was performed using the Noraxon MR3 software (Noraxon Inc., AZ, United States). Electrocardiogram contamination was removed from the EMG signals, followed by a bandwidth filter of 20–500 Hz, full-wave rectification, and smoothing (root mean square of 100 milliseconds). The average of three repetitions of the 5-s isometric hold was used for further data analysis of the 3D scapular position and EMG activity of each muscle in all nine movements. For the MVIC analysis, a marker was manually placed at the beginning and after 3 s for each repetition, and the average amplitude over 3 s between two markers for each muscle was calculated. The maximum mean of three repetitions per muscle was used for data analysis. The EMG data for each muscle recorded during the YJB exercise was subsequently expressed as the percentage of respective MVIC (%MVIC). The EMG activity levels were divided into the following categories to facilitate interpretation: low (< 20% MVIC), moderate (20%–40% MVIC), and high (>40% MVIC) (Kibler et al., 2008; Escamilla et al., 2009; Chopp-Hurley et al., 2018).

### 2.6 Statistical analysis

All data are expressed as mean and standard deviation unless otherwise stated. All statistical analyses were performed using SPSS version 26.0 for Windows (IBM Corp., NY, United States). The significance level was set to *p* < 0.05.

EMG activity and 3D scapular motion at different YJB movements were analyzed using generalized estimating equations. Significant interactions or main effects were followed by pairwise comparisons with Bonferroni correction. For these multiple comparisons, the significance level was adjusted by dividing the conventional 0.05 level by the number of comparisons performed, according to Bonferroni correction.

## 3 Results

### 3.1 Baseline characteristics of participants

**FIGURE 2:**
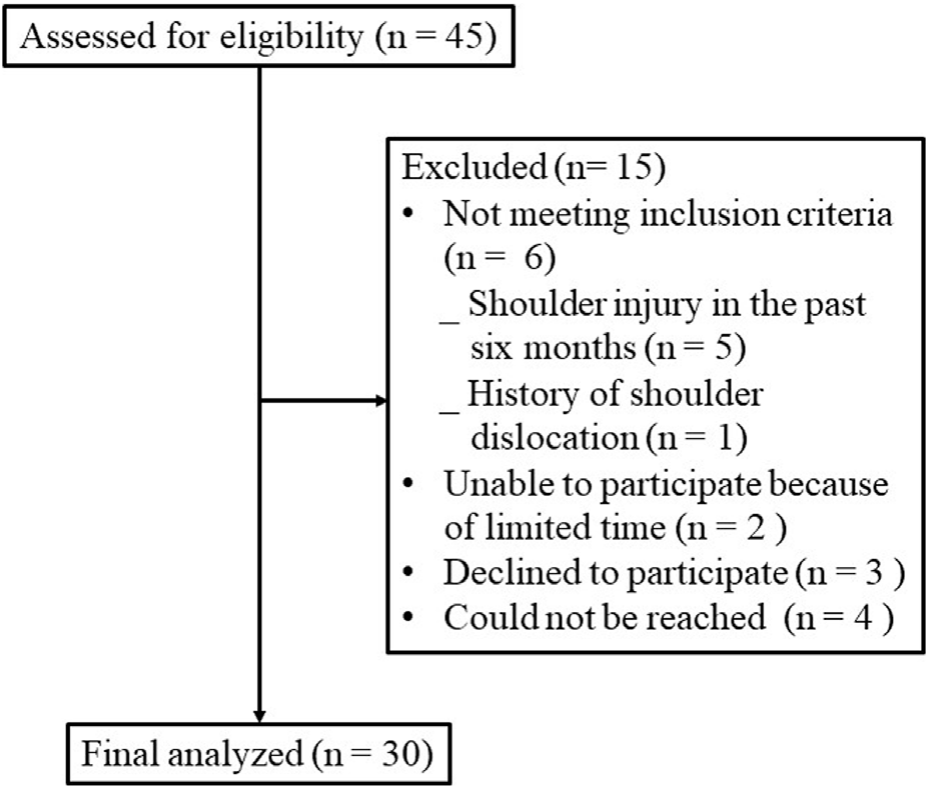
Flow of participants through the trial.

Figure 2 shows the flow of participants throughout the trial. Thirty healthy college students (men, *n* = 22; women, *n* = 8) voluntarily participated in the study. Their mean age was 20.8 ± 3.2 years, mean height was 177.4 ± 8.7 cm, mean weight was 67.8 ± 14.3 kg, mean body mass index was 21.4 ± 3.2 kg/m^2^, and mean DASH score was 2.1 ± 3.3.

### 3.2 3D kinematics

**FIGURE 3:**
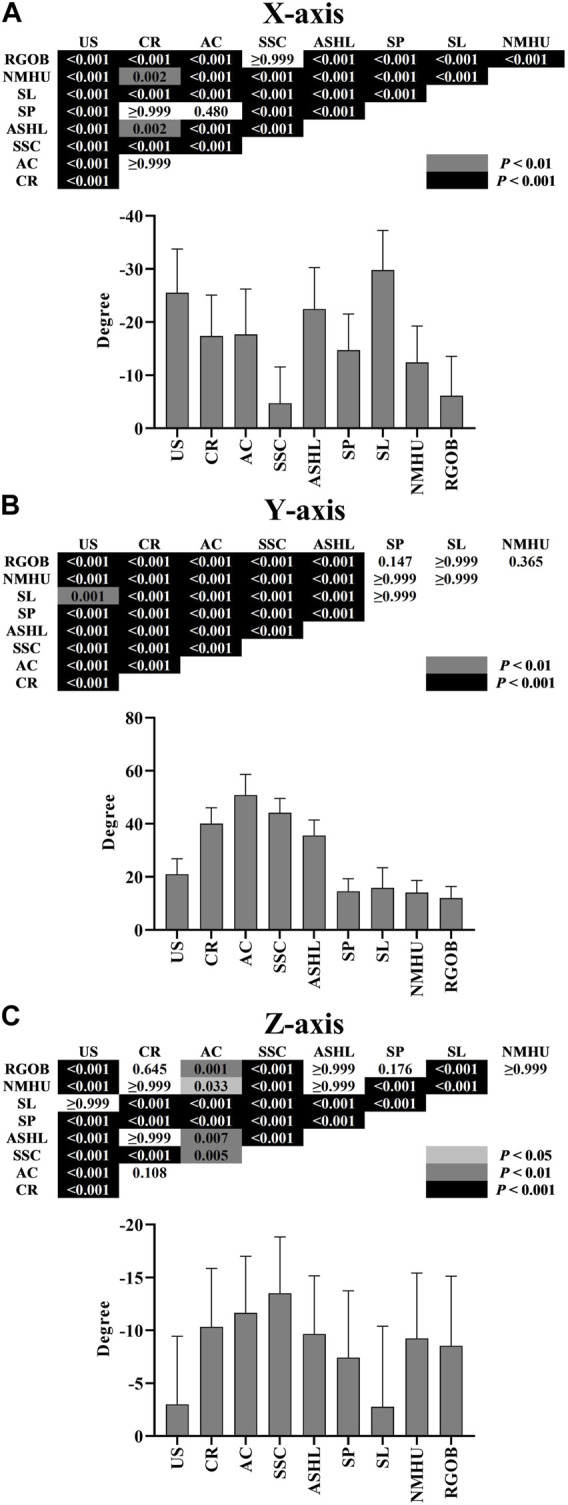
Scapular rotation (mean ± SD): upward rotation around the *X*-axis **(A)**, internal rotation around the *Y*-axis **(B)**, and anterior tilt around the *Z*-axis **(C)** during nine Yi Jin Bang movements. AC = arm crossover; ASHL = armpit support high lift; CR = column rotation; NMHU = neck massage head up; RGOB = reverse grip over back; SL = shouldering lantern; SP = shoulder press; SSC = shoulder support circle; US = upward swing.

The mean resting scapular angle with the arms relaxed at the side was 0.16° ± 5.62° of upward rotation, 31.54° ± 5.34° of internal rotation, and 12.17° ± 5.50° of anterior tilt. All nine YJB movements that resulted in scapular motions in the X-, Y-, and *Z*-axes were upward rotation, internal rotation, and anterior tilt, respectively. Compared with the mean resting scapular angle, the scapula was more upwardly rotated and less or similarly anteriorly tilted during all nine YJB movements. Column rotation, arm crossover, shoulder support circle, and armpit support high lift generated more internal rotation than the mean resting scapular angle. In contrast, the other five movements generated less internal rotation than the mean resting scapular angle. A significant interaction effect was detected between scapular rotations around the X-, Y-, and *Z*-axes and the nine movements (*p* < 0.001). Figure 3 shows the results of the pairwise comparison. The scapula was significantly less upwardly rotated during the shoulder support circle and reverse grip over back (−4.72° and -6.14°, respectively) compared with the other seven movements (−29.80° to −12.41°; *p* < 0.001). Compared with column rotation, arm crossover, shoulder press, and neck massage head up (−17.69° to −12.41°), the upward swing, armpit support high lift, and shouldering lantern generated significantly more scapular upward rotation (−29.80° to −22.46°, respectively; *p* < 0.01). The scapula was significantly more internally rotated during the upward swing, column rotation, arm crossover, shoulder support circle, and armpit support high lift (21.03°–50.84°) compared with the other four movements (12.00°–15.82°; *p* < 0.01). Compared to column rotation, arm crossover, shoulder support circle, and armpit support high lift (35.60°–50.84°), the upward swing generated significantly less scapular internal rotation (21.03°; *p* < 0.001). Additionally, the scapula was significantly less anteriorly tilted during the upward swing and shouldering lantern (−3.00° and −2.78°, respectively) compared to the other seven movements (−13.50° to −7.43°; *p* < 0.001). Compared to column rotation, arm crossover, armpit support high lift, shoulder press, neck massage head up, and reverse grip over back (−11.66° to −7.43°), the shoulder support circle generated significantly more scapular anterior tilt (−13.50°; *p* < 0.01).

### 3.3 EMG activity

**FIGURE 4:**
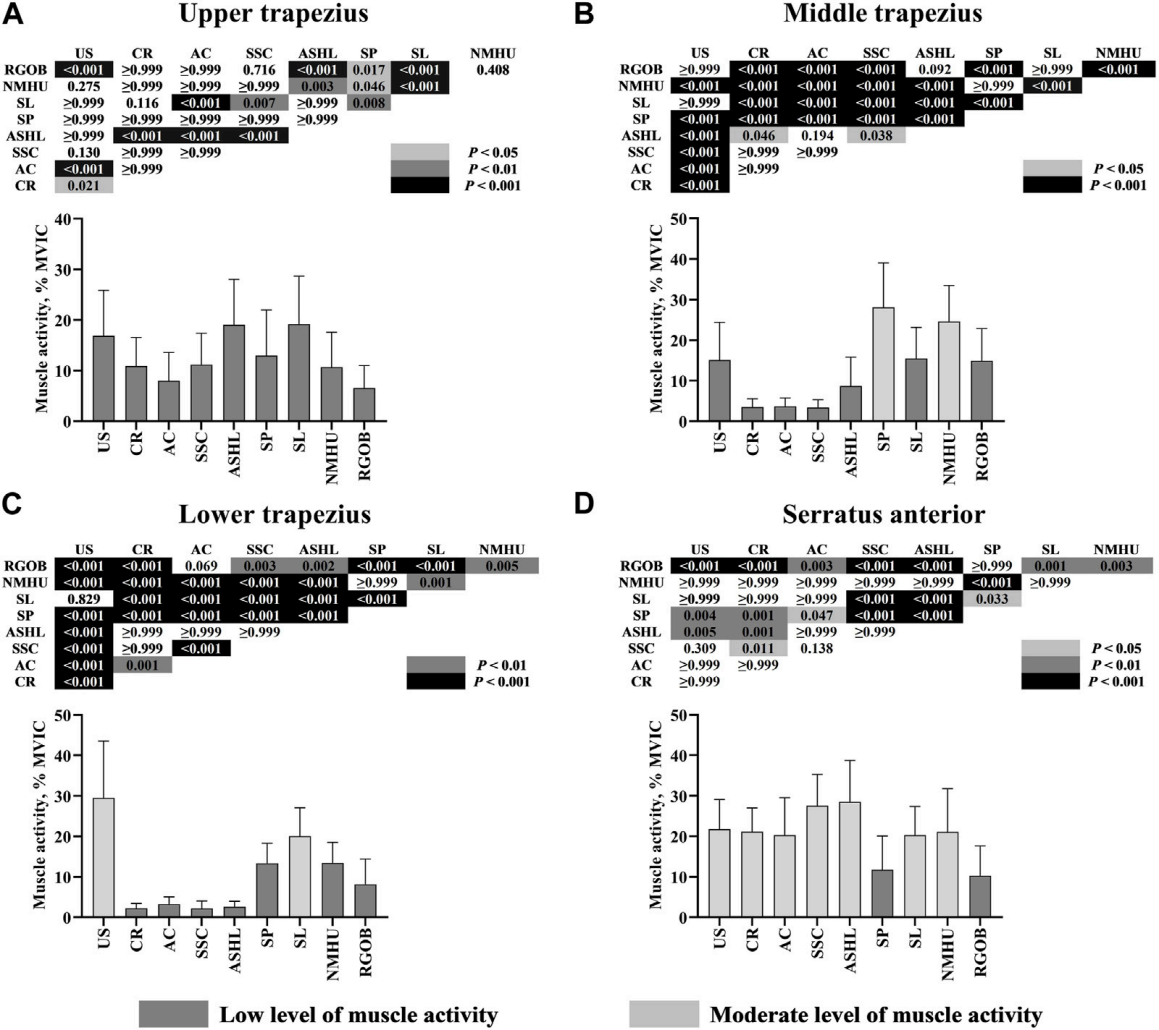
Electromyography activity (mean ± SD) of four scapulothoracic muscles: upper trapezius **(A)**, middle trapezius **(B)**, lower trapezius **(C)**, and serratus anterior **(D)** during nine Yi Jin Bang movements. AC = arm crossover; ASHL = armpit support high lift; CR = column rotation; MVIC = maximal voluntary isometric contraction; NMHU = neck massage head up; RGOB = reverse grip over back; SL = shouldering lantern; SP = shoulder press; SSC = shoulder support circle; US = upward swing.

**FIGURE 5:**
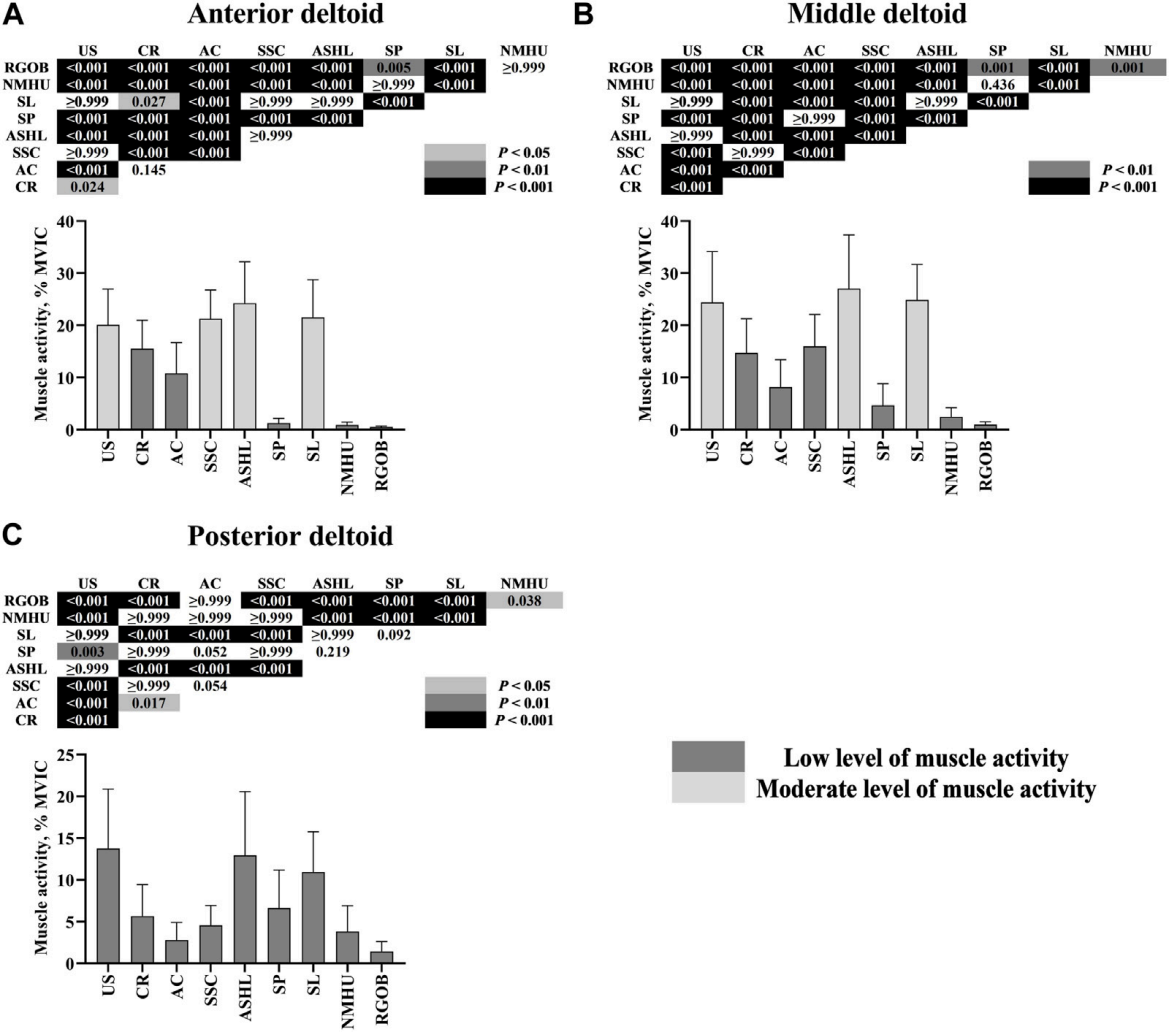
Electromyography activity (mean ± SD) of three scapulohumeral muscles: anterior deltoid **(A)**, middle deltoid **(B)**, and posterior deltoid **(C)** during nine Yi Jin Bang movements. AC = arm crossover; ASHL = armpit support high lift; CR = column rotation; MVIC = maximal voluntary isometric contraction; NMHU = neck massage head up; RGOB = reverse grip over back; SL = shouldering lantern; SP = shoulder press; SSC = shoulder support circle; US = upward swing.

A significant interaction between the seven muscles and the nine movements was found (*p* < 0.001). The results of the pairwise comparisons are shown in Figure 4; Figure 5. Overall, the YJB movements elicited low to moderate activity of the examined muscles, with no high muscle activity generated during all of the nine YJB movements. For the upper trapezius, all YJB movements resulted in low activity level (6.56%–19.19% MVIC). The middle trapezius activity was moderate during the shoulder press and neck massage head up (28.13% and 24.63% MVIC, respectively), significantly higher than the low middle trapezius activity level during the other seven movements (3.41%–15.52% MVIC; *p* < 0.001). The upward swing and shouldering lantern elicited moderate lower trapezius activity (29.50% and 20.06% MVIC, respectively), which was significantly higher than the low lower trapezius activity level during the other seven movements (2.21%–13.43% MVIC; *p* < 0.01). Only the shoulder press and reverse grip over back generated low serratus anterior activity (11.74% and 10.26% MVIC, respectively), while the other seven movements produced significantly greater serratus anterior activity and reached a moderate activity level (20.31%–28.56% MVIC; *p* < 0.05). The anterior deltoid was significantly more activated and reached a moderate activity level during the upward swing, shoulder support circle, armpit support high lift, and shouldering lantern (20.07%–24.26% MVIC) compared to the low anterior deltoid activity during the other five movements (0.52%–15.53% MVIC; *p* < 0.05). For the middle deltoid, the moderate middle deltoid activity generated during the upward swing, armpit support high lift, and shouldering lantern (24.41%–27.03% MVIC) was significantly greater than the low middle deltoid activity generated during the other six movements (0.97%–15.99% MVIC; *p* < 0.001). In addition, only low posterior deltoid activity was generated during all nine YJB movements (1.42%–13.75% MVIC).

## 4 Discussion

This study examined the 3D kinematics of the scapula and EMG activity of the scapular muscles during nine YJB movements in healthy adults. The results support our hypothesis that different YJB movements would produce variations in 3D scapular kinematics and scapular muscle activation patterns.

As already mentioned, ideal exercises for improving scapular control should enhance the activation of the middle trapezius, lower trapezius, and serratus anterior with minimal input from the upper trapezius. A moderate level of muscle activity (i.e., 20%–40% MVIC) has been considered high enough to retrain neuromuscular control of the scapula (Kibler et al., 2008). In all nine YJB movements, only the reverse grip over back elicited low activity of all four scapulothoracic muscles, while the other eight movements elicited moderate activity levels from one or two of the middle trapezius, lower trapezius, and serratus anterior and elicited a low level of activity from the upper trapezius. In addition, Reinold et al. found that the anterior deltoid and middle deltoid significantly impact superior humeral head migration and suggested that high levels of the anterior deltoid and middle deltoid activity should be minimized in most patients with shoulder pathology (Reinold et al., 2009). In our study, except for the upward swing, shoulder support circle, armpit support high lift, and shouldering lantern, which elicited moderate activity levels from the anterior deltoid and/or middle deltoid, the other five movements elicited low levels of activity from both the anterior deltoid and middle deltoid.

Several studies have evaluated the activity of scapulothoracic muscles (i.e., upper trapezius, middle trapezius, lower trapezius, and serratus anterior) during the isometric phase of shoulder rehabilitation exercises in healthy populations. The low to moderate muscle activity level required to perform YJB exercises was lower than that demonstrated in one study evaluating shoulder girdle strengthening exercises (i.e., side-lying external rotation, side-lying forward flexion, prone horizontal abduction with external rotation, and prone extension) (Cools et al., 2007) and comparable to commonly used early and middle shoulder rehabilitation exercises (i.e., inferior glide, low row, lawnmower, and robbery) reported in another study (Berckmans et al., 2020). However, because the intensity level or nature of the YJB exercise differs from other exercises, comparing the muscle activity produced by the YJB exercise found in the current study with other studies is challenging. Many clinicians recommend using exercises that require less than 20% of the patient’s MVIC in the early phase of shoulder rehabilitation (Uhl et al., 2010). Therefore, based on the scapular muscle activity levels of the YJB exercise, YJB exercises may be more suitable in the middle to the late phase of shoulder rehabilitation than in the early phase.

The upward swing, armpit support high lift, and shouldering lantern showed a greater degree of upward rotation compared to the other six YJB movements. This can be explained by the higher position of the arm during the isometric contraction phase. The upward rotation was found to be related to the position of the arm, in which greater upward rotation values were observed in exercises with a higher arm elevation (Berckmans et al., 2020). The upward swing showed lesser degrees of internal rotation than the column rotation, arm crossover, shoulder support circle, and armpit support high lift, probably due to the greater activity of the middle trapezius. Our study found that the middle trapezius activity during the upward swing was significantly higher than the middle trapezius activity generated during the column rotation, arm crossover, shoulder support circle, and armpit support high lift. This agrees with previous findings that the middle trapezius activity might be associated with the production of external scapular rotation (McClure et al., 2012). Additionally, although the shoulder press and neck massage head up elicited significantly higher middle trapezius activity than the shouldering lantern and reverse grip over back, these four movements generated similar values of internal scapular rotation. We assume this is because the shoulder press and neck massage head up involved scapular retraction exercises, resulting in external scapular rotation (Yamauchi et al., 2015). In our study, the lower trapezius activity during the upward swing and shouldering lantern were significantly higher than the other seven movements, with the scapula significantly less anteriorly tilted during the upward swing and shouldering lantern than the other seven movements. This finding supports the assertion that decreased lower trapezius activity may result in a more anterior tilt of the scapula (Berckmans et al., 2020).

The resting scapular angle in asymptomatic subjects has been reported to range from 5.4° to −5.3° of upward rotation, 26.5°–41.1° of internal rotation, and 2°–15.9° of anterior tilt, where negative values refer to downward scapular rotation (Struyf et al., 2011). This is comparable with the resting scapular angle found in the current study. Although there are some inconsistencies, the general pattern of the scapular kinematics observed in individuals with SAPS is increased internal rotation, anterior tilt, and decreased upward rotation (Oyama et al., 2010). Exercises aimed to address these dyskinesias may have the potential to restore normal scapular kinematics effectively. All YJB movements could be feasible to implement in patients with insufficient upward rotation and/or posterior tilt. However, column rotation, arm crossover, shoulder support circle, and armpit support high lift may not be suitable for patients who exhibit insufficient external rotation since these movements place the scapula in a more internal rotation position than the resting scapular internal rotation angle.

Some limitations of this study should be noted. First, this study included only healthy participants. Thus, caution should be exercised when generalizing these results. Future studies are needed to investigate the EMG activity of the scapular muscles and the 3D kinematics of the scapula during YJB exercises in patients with SAPS. Second, this study only reported the activation patterns of the superficial scapular muscle activity during YJB exercises using surface EMG. However, the activation patterns of deep scapular stabilizers and rotator cuff muscles during YJB exercises remain unknown. Future studies should also be conducted to explore the activity of the deeper-layer scapular and rotator cuff muscles during YJB exercises using fine-wire EMG. Third, the markers on the skin may not accurately capture the scapular motions because the soft tissue on the scapula is thick. We believe that we can minimize the impact of soft tissue thickness because participants in our study were fit with normal weight (mean body mass index 21.4 ± 3.2 kg/m^2^).

## 5 Conclusion

This study provided information about 3D scapular kinematics and EMG activity of the scapular stabilizer muscles during YJB exercises. Almost all YJB movements elicited moderate activity levels from one or two of the middle trapezius, lower trapezius, and serratus anterior and elicited low activity from the upper trapezius. The findings suggest that YJB exercises could be useful in the middle and later phases of shoulder rehabilitation. The column rotation, arm crossover, shoulder support circle, and armpit support high lift induced more internal scapular rotation and thus should not be recommended for patients with insufficient external rotation. This information may assist clinicians in developing treatment programs for scapular rehabilitation using YJB exercises.

## Data Availability

The raw data supporting the conclusion of this article will be made available by the authors, without undue reservation.
