# Supplementary material for: Scapular kinematics and muscle activity during Yi Jin Bang exercises
Source: Front Physiol. 2023 Jun 8;14:1169092. doi: 10.3389/fphys.2023.1169092 (PMC10285390; doi:10.3389/fphys.2023.1169092)
Supplement: Supplementary file 1 [file DataSheet1.PDF]

Upward Swing

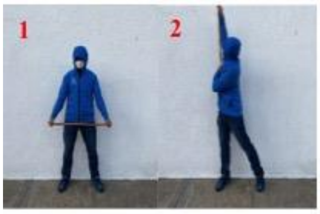

Column Rotation

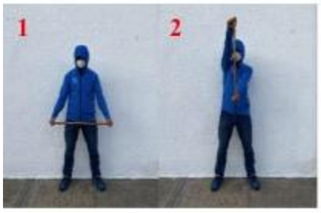

Arm Crossover

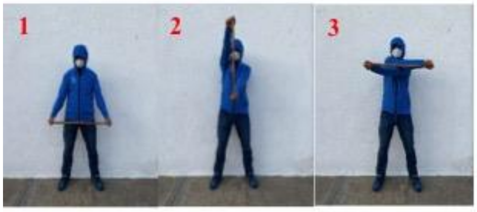

Shoulder Support Circle

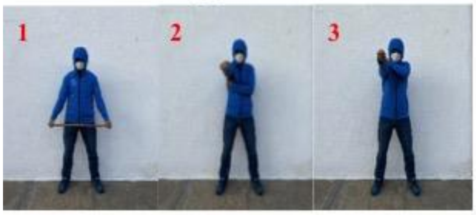

Armpit Support High Lift

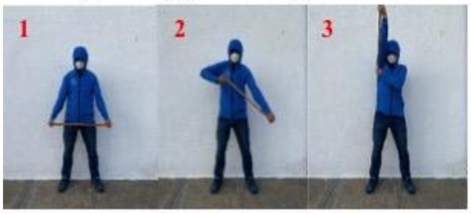

Shoulder Press

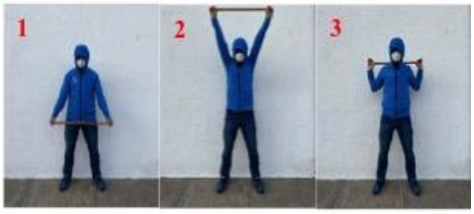

Shouldering Lantern

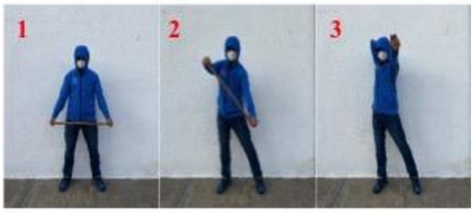

Neck Massage Head Up

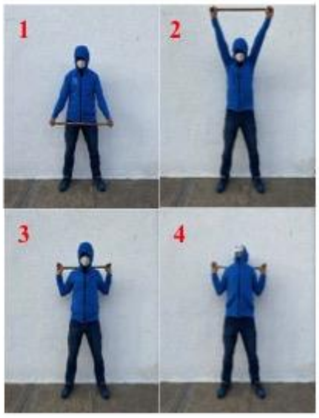

Reverse Grip Over Back

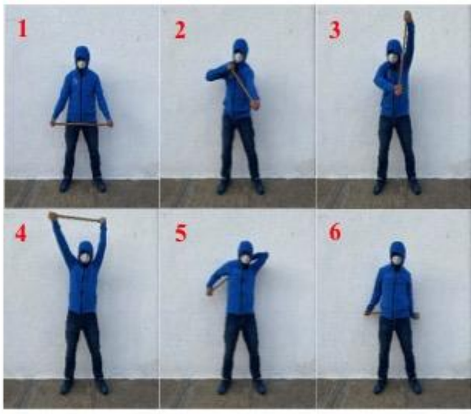

A video demonstration is available online at https://bit.ly/3td9HPJ for reviewers' reference.
